# Surgical management of penetrating pulmonary injuries

**DOI:** 10.1186/1757-7241-17-8

**Published:** 2009-02-23

**Authors:** Patrizio Petrone, Juan A Asensio

**Affiliations:** 1 Department of Surgery, University of Southern California Keck School of Medicine, Los Angeles, CA, USA; 2 Department of Surgery, University of Miami Miller School of Miami, Miami, FL, USA

## Abstract

Chest injuries were reported as early as 3000 BC in the Edwin Smith Surgical Papyrus. Ancient Greek chronicles reveal that they had anatomic knowledge of the thoracic structures. Even in the ancient world, most of the therapeutic modalities for chest wounds and traumatic pulmonary injuries were developed during wartime.

The majority of lung injuries can be managed non-operatively, but pulmonary injuries that require operative surgical intervention can be quite challenging. Recent progress in treating severe pulmonary injuries has relied on finding shorter and simpler lung-sparing techniques. The applicability of stapled pulmonary tractotomy was confirmed as a safe and valuable procedure.

Advancement in technology have revolutionized thoracic surgery and ushered in the era of video-assisted thoracoscopic surgery (VATS), providing an alternative method for accurate and direct evaluation of the lung parenchyma, mediastinum, and diaphragmatic injuries.

The aim of this article is to describe the incidence of the penetrating pulmonary injuries, the ultimate techniques used in its operative management, as well as the diagnosis, complications, and morbidity and mortality.

## Introduction

Chest injuries were reported as early as 3000 BC in the Edwin Smith Surgical Papyrus [1]. Ancient Greek chronicles reveal that they had anatomic knowledge of the thoracic structures and the position of the lungs inside the hemithoracic cavities, being proof of that the Homer's Iliad [2] with the vivid description of the death of Sarpedon.

Galen, one of the most prominent physicians of antiquity, described packing of chest wounds in gladiators with thoracic injuries [3].

Even in the ancient world, most of the therapeutic modalities for chest wounds and traumatic pulmonary injuries were developed during wartime, especially by Ambroise Paré [4], John Hunter [4], and Jean-Dominique Larrey [4].

The liberal use of thoracentesis in the management of hemothorax, the creation of the Mobile Army Surgical Hospital (MASH) units, and early evacuation from the combat zone directly to well-organized trauma centers operated under strict resuscitative protocols during World War II, and the Korean and Vietnam conflicts, have contributed to lower the mortality [5,6]. Tube thoracostomy remains the cornerstone for the treatment of traumatic injuries to the lung [7].

Recent awareness based on civilian and military experience has led to recognition that complex procedures in critically injured patients often develop hypothermia, acidosis, coagulopathy, and dysrhythmias [8-10]. Recent progress in treating severe pulmonary injuries has relied on finding shorter and simpler lung-sparing techniques [5,11]. The applicability of stapled pulmonary tractotomy was confirmed as a safe and valuable procedure [12,13], and the lung-sparing techniques are associated with an improved morbidity and mortality [14].

Advancement in technology have revolutionized thoracic surgery and ushered in the era of video-assisted thoracoscopic surgery (VATS), providing an alternative method for accurate and direct evaluation of the lung parenchyma, mediastinum, and diaphragmatic injuries, with the advantage of allowing definitive treatment of such injuries [15]. VATS also has been demonstrated to be a reliable operative therapy for complications, including post-traumatic pleural collections [16].

## Incidence

The true incidence of pulmonary injuries is unknown and difficult to estimate from the literature [17-19]. The reported incidence of pulmonary injuries in the civilian arena varies according to authors and institutions. Graham et al [20] reported 1-year experience, consisting of 373 patients sustaining penetrating pulmonary injuries. Robison et al [21] described a 13-year civilian experience in the management of pulmonary injuries in 1168 patients. Tominaga and colleagues [22] described a 7-year single institutional experience of 2934 patients sustaining both blunt and penetrating chest trauma. Recently, our group [23] described 101 patients who sustained complex penetrating pulmonary injuries. In the military arena, Zakharia et al [24] reported 1992 casualties during the Lebanon's conflict, with an incidence of 11%. Petricevic and associates [25] reported on 2547 casualties from the Balkan war experience, 16% of those sustained both blunt and penetrating chest wounds.

## Etiology

The majority of thoracic injuries requiring surgical intervention are due to penetrating mechanisms of injury such as gunshot wounds (GSW), stab wounds (SW) and shotgun wounds (SGW). Much less common are blunt thoracic injuries requiring operative intervention, but this mechanism of injury is in gradual rise from 3% before 1994 to 12% in the latter period, mostly from motor vehicle collisions [26]. Tominaga et al [22] accounted in their series 25% as blunt mechanism.

Gunshot wounds represent the major penetrating mechanism of injury for patients requiring surgical treatment, ranging from 33% to 80% of the cases [13,14,20-22], while stab wounds account for 17% to 67% of these injuries [13,14]. Other mechanisms such as impalement and shotgun wounds are reported with a lower frequency of 1% to 5% of cases [13,14].

### Classification

In 1994 the American Association for the Surgery of Trauma – Organ Injury Scaling Committee (AAST-OIS) describes the lung injury scale (Table 1) [27]. This scale facilitates clinical research and provides a common nomenclature by which trauma surgeons may describe lung injuries and their severity.

**Table 1:** American Association for the Surgery of Trauma – Organ Injury Scaling: Lung Injury [27]

**Grade^a^**	**Injury Type**	**Description^b^**
I	Contusion	Unilateral, < 1 lobe

II	Contusion	Unilateral, single lobe
	Laceration	Simple pneumothorax

III	Contusion	Unilateral, > 1 lobe
	Laceration	Persistent (> 72 hours), air leak from distal airway
	Hematoma	Non-expanding intraparenchymal

IV	Laceration	Major (segmental or lobar) air leak
	Hematoma	Expanding intraparenchymal
	Vascular	Primary branch intrapulmonary vessel disruption

V	Vascular	Hilar vessel disruption

VI	Vascular	Total, uncontained transection of pulmonary hilum

^a ^Advance one grade for multiple injuries up to grade III. Hemothorax is scored under thoracic vascular organ injury scale.

^b ^Based on most accurate assessement at autopsy, operation, or radiological study.

## Diagnosis

### Physical examination

The clinical presentation of patients sustaining penetrating pulmonary injuries ranges from hemodynamic stability to cardiopulmonary arrest [28]. Patients with penetrating pulmonary injuries may present with symptoms and signs of pneumohemothorax or an open pneumothorax with a partial loss of the chest wall, or may also present with a tension pneumothorax [28,29].

Patients with penetrating pulmonary injuries may rarely present with a pneumomediastinum upon auscultation. Hamman's Crunch – a systolic crunch – may be detected upon auscultation in these patients. Similarly, as they may also present with a pneumopericardium detected by auscultating Brichiteau's windmill bruit (bruit de moulin). Patients with penetrating pulmonary injuries may rarely present with true hemoptysis, and sometimes with symptoms and signs of associated cardiac injuries [17,18,28].

During the evaluation of these patients, the trauma surgeon must be cognizant that the thoracic cavity is composed of both right and left hemithoracic cavity as well as an anterior, posterior and superior mediastinum, as often missiles or other wounding agents may traverse one or both cavities [28,30-33]. Similarly, missile trajectories are often unpredictable and frequently create secondary missiles if they impact on hard bony structures such as the ribs, spine and sternum thus creating the potential for associated injuries and greater damage.

### Non-invasive diagnostic modalities

#### Trauma ultrasound

The Focused Assessment Sonogram for Trauma (FAST) is performed as part of the secondary survey. It diagnoses and excludes an associated cardiac injury and can also diagnose the presence of a hemothorax. On the basis of these findings, Knudson et al [34] concluded that ultrasound is a reliable modality for the diagnosis of pneumothorax, and it may serve as an adjunct or precursor to routine chest radiograph in the evaluation of injured patients.

#### Chest X-Ray (CXR)

A standard supine posteroanterior CXR is the most frequently used diagnostic modality in patients who sustain traumatic lung injury. Radiological diagnosis of traumatic pulmonary injuries is based on the presence of pneumothorax, pleural fluid collections, intrapulmonary hematomas, traumatic pneumatoceles, and pulmonary parenchymal contusions [7,17,18,20,28,29,35].

#### Computed Tomography (CT)

The most common types of abnormalities seen on CT scans include parenchymal lacerations, post-traumatic hemothorax and pneumothorax, atelectasis, subcutaneous emphysema, pneumopericardium and hemopericardium, and chest wall fractures. CT scans are also able to detect the presence of associated thoracic and mediastinal vascular injuries, as well as associated cervical spine and intra-abdominal injuries in about 30% of cases [36].

#### Electrocardiogram (EKG)

In some cases, EKG may exhibit changes caused by associated injuries, most commonly penetrating or blunt cardiac trauma consisting of findings related to myocardial injury [37,38]. However, nonspecific EKG abnormalities are more often seen, and are related to systemic factors such as pain, decreased intravascular volume, hypoxia, abnormal concentration of serum electrolytes, and changes in sympathetic or parasympathetic tone [37,38].

### Invasive diagnostic modalities

#### Thoracostomy

Chest tube placement may be diagnostic as well as therapeutic [7]. It will serve to evacuate air, evacuate and quantify blood, detect massive air leaks, and establish an indication for thoracotomy [32,33]. Drainage of gastrointestinal contents implies an esophageal [31], gastric, or intestinal injury [30].

#### Video-Assisted Thoracoscopic Surgery (VATS)

VATS has provided the trauma surgeon with an alternative method for the accurate and direct evaluation of the lung parenchyma, mediastinum, and diaphragmatic injuries [39,40], with the advantage of simultaneously allowing definitive treatment of such injuries [15]. VATS also has been demonstrated to be an accurate, safe and reliable operative therapy for complications of lung trauma, including post-traumatic pleural collections [16].

## Operative Management

### Instruments

**Figure 1:**
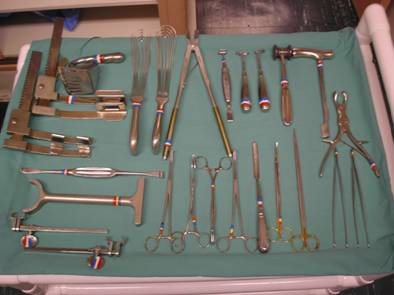
**Thoracic instrument tray**.

**Figure 2:**
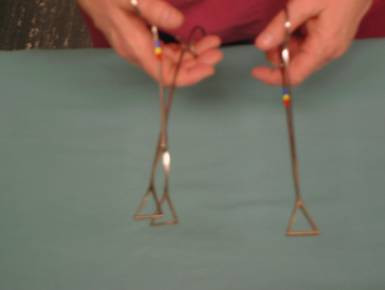
**Duval lung forceps**.

Special instruments are needed to access the thoracic cavity as well as to retract, manipulate, and surgically intervene in the thoracic structures and lungs (Figure 1 and Figure 2).

### Adjuncts

**Figure 3:**
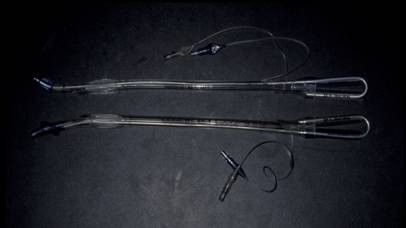
**Double lumen endotracheal tubes**.

Double lumen tubes are invaluable adjuncts in the management of penetrating pulmonary injuries (Figure 3). Although more difficult to insert by the anesthesiologist, double lumen tubes are designed to ventilate either the right or left lung selectively [41]. There are two types of double lumen tubes, one designed for the left and one designed for the right mainstem bronchus. By inflating the balloon which occludes either the right or the left mainstem bronchus, the lung can be collapsed, thus allowing the trauma surgeon to operate on a collapsed and still lung [41]. Bronchoscopy is also an invaluable adjunct when utilized intraoperatively. It can serve as a diagnostic tool by locating injured bronchi at the lobar and even segmental levels. It can also be therapeutic by removing blood within the tracheobronchial tree which tends to cause bronchospasm.

### Ventilation

The conventional ventilation method intermittently allows for a periodic inflation and deflation of the lung or high frequency jet ventilation which allows the trauma surgeon to operate on a non-moving still lung [41].

### Surgical incisions and exposures

The three most commonly used incisions in the management of penetrating cardiothoracic injuries are: the left anterolateral thoracotomy, the posterolateral thoracotomy, and median sternotomy. Each incision has its specific indications, advantages and disadvantages.

The left anterolateral thoracotomy (Spangaro's incision) is the incision of choice for the management of patients with penetrating pulmonary or cardiac injuries who arrive "in extremis". This incision is most often used in the ED for resuscitative purposes. Similarly, it is the incision of choice in patients undergoing celiotomy who deteriorate secondary to possible or unsuspected pulmonary or cardiac injuries. It can be extended across the sternum as bilateral anterolateral thoracotomies if the patient's injuries extend into the right hemithoracic cavity. This is the incision of choice in a patient who is hemodynamically unstable owing to injuries that have traversed the mediastinum or one who has sustained associated abdominal injuries. It allows full exposure of the anterior mediastinum and pericardium and both hemithoracic cavities [42-44].

The classic posterolateral thoracotomy incision is the most useful of all incisions for the management of all pulmonary injuries [42-44]. This incision is ideal for the management of thoracic injuries, such as aortic or pulmonary (left posterolateral) or pulmonary or esophageal (right posterolateral) injuries. However, it is time consuming to position the patient and can only be used if the patient is hemodynamically stable and the trauma surgeon is absolutely sure that the injury is confined to an ipsilateral hemithoracic cavity.

The median sternotomy (Duval's incision) is the incision of choice for the management of patients with associated cardiac injuries that arrive with vital signs in the operating room [42-44]. The right or left hemithoracic cavities can be accessed if the mediastinal pleura is sharply transected. This provides access to the anterior portions of either the right or left lung although exposure of the posterior aspects of the pulmonary lobes is suboptimal.

## Surgical techniques of repair and resection

The high mortality rates reported for lobectomy and pneumonectomy when performed after traumatic lung injuries, has served to develop less extensive resection techniques [5,11-14,19,21,22,26]. These techniques have been denominated 'lung-sparing techniques', and include suture pneumonorrhaphy, stapled and clamp pulmonary tractotomy with selective vessel ligation, and non-anatomic resection.

These procedures are indicated for control of hemorrhage, control of small air leaks, to preserve pulmonary tissue, and/or when the pulmonary injury is amenable to reconstruction. It is estimated that approximately 85% of all penetrating pulmonary injuries can be managed with these techniques [5,12-14].

### Suture pneumorrhaphy

The lung is stabilized with Duval lung forceps. Stay absorbable sutures are placed in the superior and inferior aspect of the wound as well as in the lateral aspects, and they are used to gently retract the edges. Very fine malleable ribbon retractors are placed to separate the wound and to provide visualization of the injured vessels which are then selectively ligated. The same is done for small bronchi. The edges of the wound are then approximated with a running locked suture [45,46].

### Stapled pulmonary tractotomy

**Figure 4:**
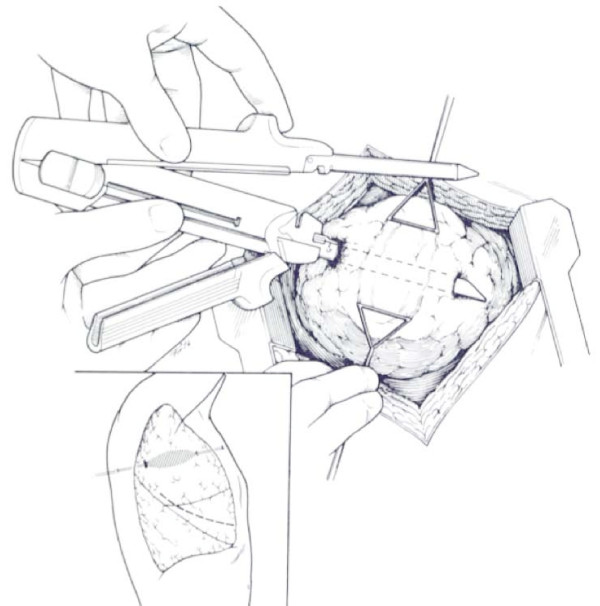
**Depicts the cavitary effect created by a missile traversing the lung**. Stapling device is placed through the orifices of entry and exit wounds.

**Figure 5:**
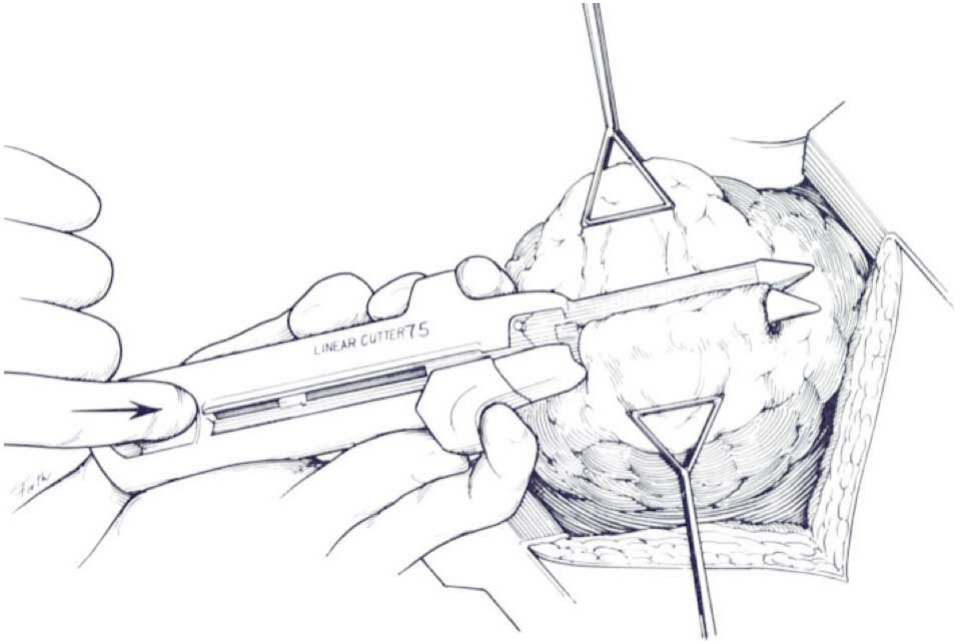
**Stapling device is closed and fired to create the tractotomy**.

**Figure 6:**
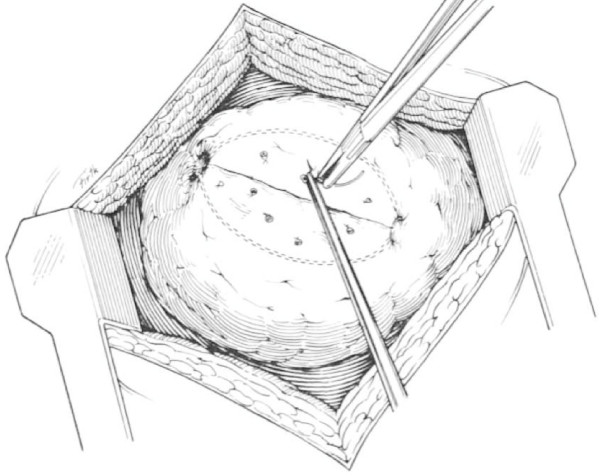
**The tract is open and the deep bleeding vessels are selectively ligated**.

Orifices of entrance and exit are defined. If need be, the overlying visceral pleura is sharply incised with Nelson scissors. A GIA 55 or 75 stapler with 3.8 mm staples is placed through the orifices of entrance and exit and fired (Figure 4 and Figure 5). This will open the tract traversed by the missile or other wounding agent effectively exposing the injured vessels and bronchi which are then selectively ligated utilizing absorbable suture (Figure 6). The lung parenchyma can then be approximated with a single running locked suture. The orifices of entry and exit are left open for the egress of air and/or blood. The integrity of the suture line is tested by having the anesthesiologist inflate the lung, and the air leaks are then detected and repaired [11,46].

### Clamp pulmonary tractotomy

The same technique as stapled pulmonary tractotomy, but instead of stapler two Crafoord-DeBakey clamps are placed through the orifices of entrance and exit and the pulmonary tissue between the clamps is sharply transected with scissors. The use of clamps may crush pulmonary tissue.

### Non-anatomic resection

This procedure is indicated when a very small and peripheral portion of a lobe or segment is devitalized. The area of resection is stabilized between Duval lung forceps and a stapler is fired across, thus resecting the injured portion of the lung. The staple line may be over sewn with a running locked suture, although this is not generally necessary. Non-anatomic resection can also be complex and require resection of major segments with complex reconstruction.

## Resectional procedures

Resectional procedures include formal lobectomy and formal pneumonectomy. These procedures are indicated for control of hemorrhage, resection of devitalized or destroyed pulmonary tissue, control of major air leaks not amenable to repair, and control of life-threatening hemorrhage [45,46].

### Formal lobectomy

To perform a lobectomy the fissure must be separated. In the case of the right lung the oblique fissure separates the upper and middle lobe from the lower lobe while the horizontal fissure separates the upper lobe from the middle lobe. In the left lung the oblique fissure divides the left upper from the left lower lobe. The lingula of the left upper lobe corresponds to the middle lobe on the right, but it is fused with the upper lobe in most cases [45,46].

Vascular dissection should be initiated extrapleurally at the hilum through a perivascular plane to find the major pulmonary vessels. Vascular dissection in the fissures identifies the lobar vessels. Transection of the inferior pulmonary ligament distally will allow greater mobility of the lower lobes of both lungs. All pulmonary vessels whether they be the main lobar vessels or segmental vessels can be ligated in continuity and transfixed with non-absorbable sutures. Alternatively, they may be stapled or may also be over sewn [45,46].

The bronchi, whether they are the main, lobar or segmental bronchi should be stapled and transected. Bronchi may also be transected utilizing Sarot lung clamps and sutured with 4-0 Tev-Dek synthetic sutures. The suture technique involves clamping the bronchus distal to the intended point of transection. The bronchus is cut transversely for 4–5 mm, and the cut end is sutured, and should be tied very carefully to avoid cutting or unnecessarily devascularization. After placement of two sutures, the cut end is extended and additional sutures are placed 2–3 mm apart. While for a main bronchus, seldom are more than six sutures required, for a lobar bronchus three to four sutures are usually enough. Too many sutures devascularized the transected bronchus. After closure is complete, the suture line is tested, and additional sutures are placed if there is an air leak detected. To prevent lung torsion the remaining lobes are pexed to the thoracic wall [45,46].

### Pneumonectomy

#### A) Right Pneumonectomy

A thorough exploration of the right hemithoracic cavity is carried out. The azygous vein is identified, and the right pulmonary hilum is located. Utilizing a meticulous combination of sharp and blunt dissection the right main pulmonary artery is identified and encircled with a vessel loop. The right inferior pulmonary ligament is sharply transected. Both superior and inferior pulmonary veins are identified and encircled with a vessel loops. All vessels may be either ligated in continuity or stapled individually. The right mainstem bronchus is then identified and encircled. The trauma surgeon must be careful not to apply undue traction to avoid tearing subcarinal structures. The bronchus is then transected [45,46].

#### B) Left Pneumonectomy

The same steps as the right pneumonectomy are taken, paying special attention with the phrenic, vagus and left recurrent laryngeal nerves which are identified and preserved, and the left pulmonary hilum is located [45,46].

## Morbidity

The most common intraoperative complication is heart failure, while the physiological post-operative complications include right ventricular failure, pulmonary artery hypertension, and "run-away" pulmonary artery hypertension.

The most common technical complications include lung hernia, lung torsion, bronchopleural fistulas, arteriovenous fistulas, bronchial stump leaks, bronchial stump blow-outs, bronchial stenosis, empyema, and lung abscess. These complications will often require surgical reintervention. Fortunately, they are infrequent.

## Mortality

The estimated mortality for these procedures is very variable. The overall mortality rate reported in the literature for patients with traumatic pulmonary injuries ranges from 1.7% to 37%. For stapled procedures the mortality is 10%, for non-anatomic resections is 20%, for lobectomies it can range from 30% to 50%, and for pneumonectomies the mortality rate is between 50% to 100% [5,12,14,22,26].

## Conclusion

Pulmonary injuries requiring thoracotomy are uncommon even in busy urban trauma centers. Simpler surgical techniques are frequently used for their management. Stapled pulmonary tractotomy has become the most frequently used lung sparing technique, and can manage 85% of all pulmonary injuries requiring surgical interventions. Despite recent advances, pulmonary injuries requiring resective procedures are marked by high morbidity and mortality.

## Competing interests

The authors declare that they have no competing interests.

## Authors' contributions

PP drafted the manuscript. PP and JAA critically revised the manuscript. PP and JAA have read and approved the final manuscript.
